# Tmprss3 is expressed in several cell types of the inner ear including type II but hardly in type I spiral ganglion neurons

**DOI:** 10.3389/fncel.2025.1690227

**Published:** 2025-11-12

**Authors:** Ramil Arora, Lucie Pifková, André Ulrich Deutschmann, Ellen Reisinger

**Affiliations:** 1 Gene therapy for hearing impairment group, Department for Otolaryngology – Head & Neck Surgery, University Hospital and Faculty of Medicine, University of Tübingen, Tübingen, Germany; 2 Gene and RNA Therapy Center (GRTC), University of Tübingen, Tübingen, Germany

**Keywords:** organ of Corti, inner hair cells, outer hair cells, stereocilia bundle, hearing impairment, cochlear implant, spiral limbus, extracellular serine protease

## Abstract

Mutations in the *transmembrane serine protease 3* gene (*TMPRSS3*) cause non-syndromic hearing impairment, with congenital (DFNB10) or late childhood onset (DFNB8). In some reports, these patients were found to have lower speech comprehension scores with cochlear implants (CIs) compared to CI users with other etiologies. Since CIs electrically stimulate spiral ganglion neurons (SGNs) to activate the auditory pathway, TMPRSS3 deficiency was presumed to cause a dysfunction or degeneration of these cells, of which type I SGNs form the predominant group. Here, we revisited the expression pattern of *Tmprss3* in the developing and mature mouse inner ear on mRNA level with quantitative few-cell PCR and RNAscope, and on protein level with immunohistochemistry with an anti-TMPRSS3 antibody validated on knock-out tissue. In the organ of Corti, we demonstrate expression of *Tmprss3* in inner and outer hair cells, particularly in the stereocilia, and in pillar cells. Furthermore, expression of this gene in root cells of the lateral wall close to the stria vascularis indicates a potential function in K^+^ recycling, and expression in the spiral limbus may be linked to the generation of the tectorial membrane. Within Rosenthal’s canal, in immature tissue, *Tmprss3* was diffusely expressed in all SGNs, but in the mature ear, in type I SGNs we found only minor mRNA amounts with qPCR, RNAscope, and no specific immunolabeling. In contrast, in type II SGNs *Tmprss3* expression is enhanced during maturation. We hypothesize that the background levels of *Tmprss3* expression in type I SGNs are not directly responsible for the vitality of these neurons, and that indirect effects, like signaling cascades dependent on TMPRSS3 in other cell types, are crucial for type I SGN function and survival.

## Introduction

Over 150 genes are known to be associated with non-syndromic hearing loss (NSHL), and more than half of these genes follow an autosomal recessive pattern of inheritance (Walls WD, Azaiez H, Smith RJH. Hereditary Hearing Loss Homepage^1^. Aug 2025). One among them, responsible for both, childhood-onset progressive (DFNB8) and congenital/prelingual (DFNB10) forms of non-syndromic hearing loss is *TMPRSS3*, which encodes a transmembrane protein with extracellular serine protease domain (Scott et al., 2001). To date, neither the targets of this protease have been identified, nor the precise role of TMPRSS3 in the cochlea. Guipponi et al. (2002) reported that the co-expression of TMPRSS3 with the amiloride-sensitive epithelial sodium channel (ENaC) resulted in a ∼6-fold increase in ENaC conductivity. However, individuals with ENaC mutations do not experience hearing loss, which rules out ENaC as a key target for the observed pathomechanism (Peters et al., 2006). Molina et al. (2013) observed a selective lack of large-conductance Ca^2+^-activated K^+^ channel KCNMA1 (BK) in TMPRSS3-deficient ears, but since KCNMA1 deficiency causes delayed hearing impairment compared to TMPRSS3-deficiency, a major role for a BK-TMRPSS3 interaction seems unlikely (Rüttiger et al., 2004).

In two cohorts of cochlear implant (CI) candidates including all age groups, DFNB8/10 accounted to 4% and 10% of the solved cases, respectively (Tropitzsch et al., 2022; Seligman et al., 2022). In these studies, the DFNB8 cases with postlingual progressive hearing impairment outnumbered the congenital/prelingual DFNB10. While DFNB10 patients mostly receive a CI in early childhood with satisfying outcome, DFNB8 patients are implanted typically in adulthood. For the latter, the duration of hearing impairment until cochlear implantation varies, since some patients are implanted as soon as their high frequency thresholds reach the criteria for CI and these are typically benefiting from electric acoustic stimulation (EAS), while others are implanted only after substantial low frequency hearing loss occurs. Eppsteiner et al. (2012) reported two DFNB8 patients who did not benefit from cochlear implantation, which was at least partially attributed to the extended deafening time of >30 years before treatment in these cases. Whether CI performance in DFNB8/10 patients is in general similar or below average compared to other etiologies is currently intense subject of debate: While some studies reported DFNB8/10 patients to have speech comprehension levels comparable to patients with other etiologies (Weegerink et al., 2011; Miyagawa et al., 2015; Holder et al., 2021; Carlson et al., 2023), four studies unraveled variable outcomes (Lee et al., 2020, 2023; Cottrell et al., 2023; Colbert et al., 2024), and two studies found below-average speech perception (Shearer et al., 2017; Tropitzsch et al., 2023). In one recent study, nine DFNB8/10 patients with CI recognized on average 30% of monosyllable words, while the median of other CI users reached 70% (Tropitzsch et al., 2023). Shearer et al. (2018) found electrical stimulation of SGNs in DFNB8/10 patients to result in on average smaller electrical responses compared to patients with other forms of deafness, indicating a loss of function of SGNs. These observations led to the “spiral ganglion hypothesis,” proposing that CI performance and speech perception test results are poorer for individuals carrying deleterious mutations in genes that discriminately affect the health and function of the SGNs than for individuals with mutations affecting the sensory otic epithelium of the cochlea (Eppsteiner et al., 2012; Shearer et al., 2017). Therefore, the loss of TMPRSS3 function, which impairs SGN health, is considered a plausible explanation for the low electrical excitability of SGNs. Contradicting the hypothesis that SGN function in DFNB8/10 patients decays over time, auditory thresholds or phoneme recognition were observed to persist in a longitudinal study for up to 17 years post implantation (Fehrmann et al., 2023).

In mice, both immunohistochemistry and *in situ* hybridization experiments found *Tmprss3* expression in the organ of Corti and in the majority of SGNs (Guipponi et al., 2002, 2008). In a mouse model with a premature stop codon in *Tmprss3* (Y260X), hair cells decayed around the onset of hearing, and a loss of more than half of SGN cell bodies was observed between 90 and 180 days of life (Fasquelle et al., 2011). The loss of SGNs can be a direct consequence of the loss of hair cells or their synapses, but in these cases, a decay in cell bodies in the Rosenthal’s canal is typically observed past 1 year after loss of synaptic input (Zilberstein et al., 2012; Fernandez et al., 2015; Stalmann et al., 2021).

Recently, single cell RNAseq revealed no or low mRNA expression of *Tmprss3* mRNA in SGNs, challenging the hypothesis that TMPRSS3 deficiency in SGNs is a major contributor to the observed pathomechanism (Chen et al., 2022; Shrestha et al., 2018; Sun et al., 2018). Here, we carefully analyzed cell-type specific expression levels of *Tmprss3* on mRNA and protein level *in situ* in the murine cochlea with a special focus on the cell types in Rosenthal’s canal, with the aim of providing a solid data set for understanding the role of TMPRSS3 in the cochlea.

## Materials and methods

### Animals

Animal care and use were reviewed and approved by the University of Tübingen’s Veterinary Care Unit, and the Animal Care and Ethics Committee of the regional board of the Federal State Government of Baden-Württemberg, Germany. All experiments were performed in accordance with the European Union Directive 2010/63/EU, which addresses the protection of animals used for experimental and other scientific purposes. The animals were kept in standardized cages under a 12/12-h light/dark cycle (lights on from 6 AM to 6 PM) in a controlled temperature and humidity environment with access to food and water *ad libitum*. Experiments were performed on tissue from C57BL/6N animals ranging in age from postnatal days 7 to 32, which were bred in our animal facility. Mice aged P14 to P32 were anesthetized by CO_2_ before decapitation. CO_2_ was introduced at a rate of approximately 30% of chamber volume per minute (i.e., about 6500 mL/min), according to the AVMA Guidelines for the Euthanasia of Animals: 2020 Edition.

To verify that the TMPRSS3 antibody binds its target specifically, immunohistochemistry was performed on tissue of *Tmprss3* knock-out (*Tmprss3*^–/–^) mice. The mouse strain used for this research project, B6;129S5-Tmprss3^tm1Lex/^Mmucd, RRID:MMRRC_032680-UCD, was obtained from the Mutant Mouse Resource and Research Center (MMRRC) at University of California at Davis, an NIH-funded strain repository, and was donated to the MMRRC by Genentech, Inc., (Tang et al., 2010). The mouse line was generated by homologous recombination in embryonic stem cells to replace exon 1 with a LacZ/Neo^*R*^ cassette. For genotyping we performed PCR using a combination of 3 primers, 5′-ACAGCCTTAACTCTCCACG-3′, 5′-GCAGCGCATCGCCTTCTATC-3′ and 5′-TTCTAGGACTTTGCTATGACC-3′. Homozygous and heterozygous littermates were sacrificed at the age of postnatal day 10 by decapitation without anesthesia. In this study, we did not use *Tmprss3*^–/–^ animals older than P10, but we infer from the *Tmprss3*^*Y*260*X/Y*260*X*^ animals from Fasquelle et al. (2011) that inner and outer hair cells develop morphologically normal but are lost between P12 and P14. Preliminary data from our *Tmprss3*^–/–^ animals confirm profound deafness in otherwise healthy adult animals, similar as shown for *Tmprss3*^*Y*260*X/Y*260*X*^ animals (Fasquelle et al., 2011).

### RNA isolation, reverse transcription and qPCR

#### Brainstem RNA isolation and reverse transcription

Total RNA was isolated from brainstem pieces of two mice per sample using TRIzol™ Reagent (15596026, Thermo Fisher Scientific) for phase separation according to the manufacturer’s protocol. In brief, brainstem tissue pieces were lysed in 800 μL TRIzol™ reagent, using a homogenizer (885480-0020, Kimble) and by passing them ≥3 times through a 30G needle to obtain a clear solution. Phase separation was performed by adding 160 μL chloroform (102445, Merck), thoroughly shaking the mixture, and centrifuging for 15 min at 4 °C. The upper aqueous phase containing the RNA was precipitated overnight with 400 μL isopropanol (59298, Merck) in the presence of 20 μg Glycogen (10814010, Thermo Fisher Scientific) at −20 °C. The RNA was pelleted, washed with 75% Ethanol (1HPH.1, Carl Roth) and solubilized in 15 μL Nuclease-free water (R0582, Thermo Fisher Scientific) for 10 min at 55 °C. The isolated RNA was stored at −80 °C for later use.

Reverse transcription was carried out using the Genaxxon biosciences Scriptase RT - cDNA Synthesis kit (Genaxxon biosciences, M3134) in a 20 μL reaction volume according to the manufacturer’s protocol. To break the secondary structure of the mRNA, the following mixture was incubated at 65 °C for 5 min: 10 μL of RNA, 2.5 μM Oligo (dT) Primer, 1.5 μM Random Hexamer, dNTPs (500 μM each). cDNA synthesis was started by adding 1× Scriptase RT Buffer, 5 mM DTT, 40 units RNase Inhibitor and 200 units Scriptase RT, the mixture was incubated at 37 °C for 10 min, 42 °C for 1 h, and the reaction was halted by heating to 70 °C for 5 min. The cDNA was ethanol precipitated overnight at −20 °C in the presence of 20 μg Glycogen (10814010, Thermo Fisher Scientific) and 0.1× the initial volume 3M Sodium Acetate (6773.2, Carl Roth). The cDNA was then pelleted by centrifugation, washed with 75% Ethanol (1HPH.1, Carl Roth) and solubilized in 50 μL Nuclease-free water (R0582, Thermo Fisher Scientific) and stored at −20 °C.

#### Few-cell RNA isolation and reverse transcription

The cytoplasmic content of few (3–12) cells per sample of the mouse inner ear at postnatal day P14-19 was sucked into a glass capillary on a patch-clamp setup under microscopic control. The capillary was pre-filled with about 8 μL pipette solution containing 140 mM KCl (P9541, Merck), 5 mM HEPES (H3375, Merck), 5 mM EGTA (03777, Merck), 3 mM MgCl_2_ (442611, Merck) (pH 7.3) and 40 units of RNase Inhibitor. After aspirating the cytosol, the content of the pipette was expelled into the PCR reaction tube by air pressure after gently breaking the pipette tip. Reverse transcription was carried out using the Genaxxon biosciences Scriptase RT - cDNA Synthesis kit (Genaxxon biosciences, M3134) in a 15 μL reaction volume as described above, but with 2.5 μM Oligo (dT) Primer and 2.5 μM Random Hexamer, and with 2-h incubation at 42 °C. After the reaction, the cDNA was ethanol precipitated overnight at −20 °C in the presence of 10 μg Glycogen (10814010, Thermo Fisher Scientific), 10 μg of Carrier-iRNA (C078, Top-Bio), and 0.1× the initial volume 3M Sodium Acetate (6773.2, Carl Roth). The cDNA was then pelleted for 60 min at 21,100 × g at 4 °C, washed with 70% Ethanol (1HPH.1, Carl Roth), solubilized in 6 μL Nuclease-free water (R0582, Thermo Fisher Scientific) and stored at −20 °C.

#### qPCR

Quantitative PCR was performed using the standard amplification protocol in a LightCycler 480 (Roche) in a 25 μL reaction volume consisting of 1× ProbeMasterMix No ROX (M3045, Genaxxon biosciences), 1× TaqMan Probe (*NF200*: Mm01191456_m1, *Tmprss3*: Mm00453694_m1, TATA box binding protein (*Tbp*): Mm00446973_m1, and tyrosine hydroxylase (*Th*): Mm00447557_m1; all Thermo Fisher Scientific), and 1 μL of cDNA solution. All qPCRs were performed in technical triplicates.

#### Data analysis

**FIGURE 1:**
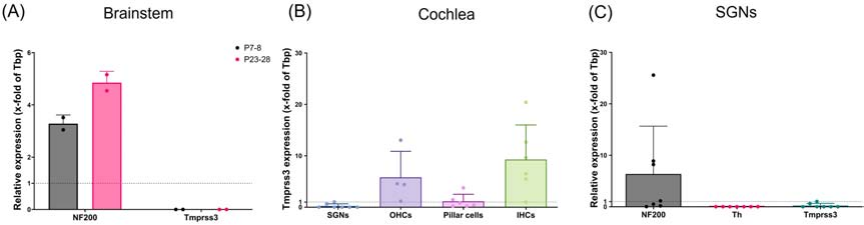
*Tmprss3* mRNA quantification with qPCR. **(A)** In the brainstem of mice at P7-8 (gray bars) or P23-28 (pink bars), NF200 was used as neuronal marker; no *Tmprss3* mRNA was detected. 2 animals per data point; data points = mean of technical triplicates; Mean ± standard deviation (SD), *N* = 4 animals per age group. **(B)** *Tmprss3* mRNA quantification in indicated cells of the cochlea at P14-P19, **(C)** quantification of *NF200*, *Th* and *Tmprss3* mRNA in SGNs at P14-P19 (each data point represents a group of 3–12 cells, *N* = 6).

Raw crossing point (Cp) values, where TaqMan fluorescence reaches a threshold, were exported and processed in MS-Excel. Samples without meaningful amplification for the reference housekeeping gene, *Tbp*, were excluded. Cp values were averaged for the technical triplicates. For each included sample, Delta Cp (ΔCp) values were calculated by subtracting the Cp value of *Tbp* from the Cp value of the target, *Tmprss3*, i.e., ΔCp = Cp target - Cp reference. Finally, relative expression (to *Tbp*) was obtained by calculating the 2^–Δ^^*Cp*^ value. For those samples in which no signal of the target was detected, the relative expression was set to zero. The relative expression obtained was plotted using *GraphPad Prism* in Figure 1.

### RNAscope and immunohistochemistry on cryosections

For RNAscope combined with immunostaining performed on cryosections, the cochleae were fixed for 24 h at 4 °C in 4% formaldehyde (P733.1, Roth) diluted in PBS (18912014, Thermo Fisher Scientific) and washed with PBS thrice. Next, the cochleae were decalcified in 200 mM EDTA, pH 7.4 (34103, Merck) at 4 °C. The length of incubation depended on the age of the animals: for P8 3 h, P14 8 h and cochleae from ≥P28 animals old were incubated for 24 h. After decalcification, the cochleae were washed with PBS thrice and incubated in 25% Sucrose (S0389, Merck) in PBS for overnight at 4°C on a test-tube rotator. The cochleae were embedded in Tissue-Tek O.C.T. (4583, Sakura) and stored at −20 °C until cryosectioning into 16 μm thick sections using a Cryostat (Leica CM3050 S) with cryostat temperature −30 °C. Sections were transferred on SuperFrost^®^ Plus slides (03-0060, R. Langenbrinck GmbH) and stored at −80 °C until staining.

RNAscope staining was performed using the RNAscope Multiplex Fluorescent Reagent Kit v2 (323100, ACD Bio-Techne). The sections were outlined with Super PAP Pen Liquid Blocker (N71312-N, Science services) and the cryosections were rehydrated in PBS for 5 min, post-fixed with 4% formaldehyde at room temperature for 10 min and washed with PBS, followed by incubation with RNAscope Hydrogen Peroxide (322381, ACD Bio-Techne) for 10 min at room temperature and washing with PBS. The following steps were performed at 40 °C in a humidified heating oven: (1) RNAscope Protease III (322381, ACD Bio-Techne) for 1 h and washing with PBS (2) RNAscope Probe Mm-*Tmprss3*-C1 (553861, ACD Bio-Techne), or for detecting mRNA of *potassium inwardly rectifying channel subfamily J member* 10 (*Kcnj10*) the probe Mm-*Kcnj10*-C2 (458831, ACD Bio-Techne) diluted 1:50 in probe Mm-*Tmprss3*-C1 for 2 h and washing with 1× RNAscope Wash Buffer (310091, ACD Bio-Techne) for 5 min, twice. The slides were kept immersed in 5× SSC buffer overnight at RT. Subsequently, the slides were washed in 1× RNAscope Wash Buffer (used for all subsequent washing steps), and 3 amplification steps were performed using RNAscope Amp 1, Amp 2 and Amp 3 (323110, ACD Bio-Techne) and followed by washing after each step. Amp 1 and 2 were incubated for 30 min at 40 °C, Amp 3 was incubated for 15 min at 40 °C.

The sections were then incubated with RNAscope HRP-C1 (323100, ACD Bio-Techne) for 15 min at 40 °C, washed, and then incubated for 30 min at 40 °C with TSA Vivid Fluorophore 570 (323272, ACD Bio-Techne) diluted 1:1500 in RNAscope TSA Buffer (322810, ACD Bio-Techne), and washed. After that the samples were incubated with RNAscope HRP blocker (323100, ACD Bio-Techne) at 40 °C for 15 min and washed. In case of using the probe Mm-*Kcnj10*-C2, the procedure was repeated for HRP-C2 and TSA Vivid Fluorophore 650.

For immunostaining, the samples were blocked for 1 h at room temperature with DSDB, composed of 17% Donkey serum (D9663, Sigma Aldrich), 0.3% Triton X-100 (T8787, Sigma-Aldrich), 20 mM Phosphate buffer pH 7.4 (Na_2_HPO_4_: S9390, Merck, and NaH_2_PO_4_: 71496, Merck), and 0.45 M NaCl (S7653, Sigma-Aldrich) in H_2_O, and then incubated with primary antibodies (TMPRSS3, 1:100, Abcam, #AB167160; Calretinin, 1:200, Swant, CG1; tyrosine hydroxylase (Th), 1:100, Merck, 657012-100UL) diluted in DSDB at 4 °C overnight. After that the samples were washed for 5 min thrice with PBS and incubated in DSDB with secondary antibodies (Donkey anti-rb IgG Alexa Fluor488, 1:200, 711-546-152, Jackson ImmunoResearch; Donkey anti-gt IgG Alexa Fluor488, 1:200, A11055, Invitrogen; Donkey anti-rb IgG Alexa Fluor647, 1:800, 711-606-152, Jackson ImmunoResearch; Donkey anti-gt IgG Alexa Fluor647, 1:400, A32849, Invitrogen) for 1 h at RT, followed by washing for 5 min thrice with PBS. Sections were then incubated with RNAscope DAPI (320858, ACD Biotechne) for 1 min at RT and mounted with Mowiol^®^ (0713.1, Roth) in conjunction with the anti-bleaching agent DABCO (0718.1, Roth) and stored at 4° C until imaging.

### RNAscope and immunohistochemistry on wholemount organs of Corti

For RNAscope combined with immunostaining of wholemounts, we used apical tips of organs of Corti dissected from cochleae after 24 h of fixation. The organs of Corti were washed with 0.1% Triton-X 100 (Merck) in PBS (PBS-T). RNAscope pretreatment with RNAscope Hydrogen Peroxide and Protease III was performed as described for cryosections, with using PBS-T for washing instead of PBS. RNAscope probe Mm-*Tmprss3*-C1 was incubated at 40 °C overnight, followed by post-fixation for 10 min at RT with 4% formaldehyde and washing with RNAscope Wash Buffer. The amplification steps, incubation with HRP-C1, TSA Vivid Fluorophore 570 and HRP blocker and immunostaining were performed as described for cryosections.

### Microscopy and image processing

**FIGURE 2:**
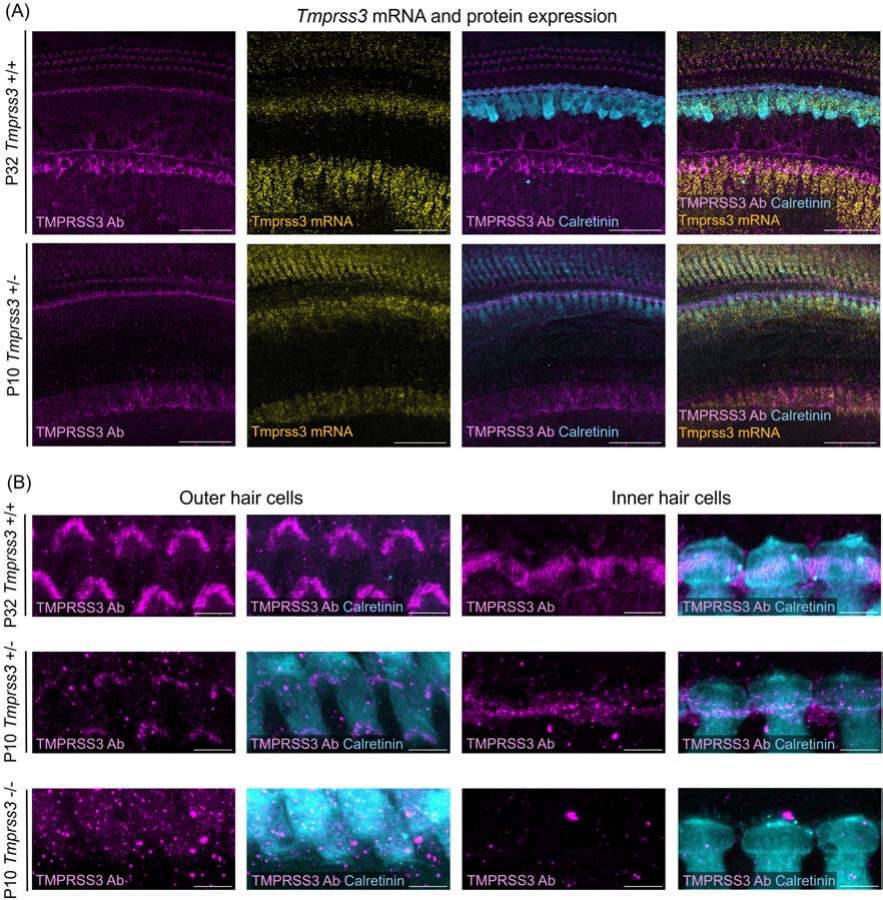
*Tmprss3* expression in wholemount organs of Corti. **(A)** Representative images of *Tmprss3* mRNA and protein expression in the apical part of the organ of Corti of a P32 wildtype (*Tmprss3*^+/+^) and a P10 heterozygous (*Tmprss3*^ + ⁣/−^) mouse. The organs of Corti are immunostained with an anti-TMPRSS3 antibody (TMPRSS3 Ab, magenta) and the hair cell marker calretinin (blue). *Tmprss3* mRNA is labeled with an RNAscope probe (yellow). TMPRSS3 protein is localized in hair bundles of inner and outer hair cells and in the spiral limbus, while *Tmprss3* mRNA is expressed in the corresponding cell bodies. Scale bars: 50 μm. **(B)** High magnification of immunolabeling for TMPRSS3 (magenta) and calretinin (blue), depicting the stereocilia of outer (left) and inner hair cells (right), indicating strong presence of TMPRSS3 in stereocilia of mature hair cells (upper panel) and weaker staining of immature hair cells of *Tmprss3*^ + ⁣/−^ mice (middle panel). Lower panel: control of immunostaining performed on homozygous knock-out (*Tmprss3*^–/–^) tissue at P10, where no immunolabeling of stereocilia occured. Scale bars: 5 μm.

Samples were imaged with a STELLARIS 5 confocal microscope (Leica Microsystems CMS, Mannheim, Germany) using a 10× air objective (numerical aperture: 0.30), a 20× glycerol immersion objective (numerical aperture: 0.75), a 40× glycerol immersion objective (numerical aperture: 1.25) and a 63× glycerol immersion objective (numerical aperture: 1.30). Z-stacks were captured with a resolution of 1024 × 512 or 1024 × 1024 pixel and a Z-step size of 1.04 μm for 20× objective, 0.36 μm for 40× objective and 0.26–0.36 μm for 63× objective. Intensity and gain were set for the wildtype controls and then used for knock-out mice of the corresponding age group, or in case of comparing developmental stages kept the same within all 3 age groups (with exception of images in Figure 2A). Maximum projections of Z-stacks were done in *ImageJ*.

RNAscope punctae per cell were counted using *ImageJ*. According to the labeling with either calretinin (Calret +) or tyrosine hydroxylase (Th +), a maximum projection from exactly those optical sections covering one cell was made and used to outline the cell borders. In the *Tmprss3* RNAscope channel of the same maximum projection, a Difference of Gaussian (DoG) algorithm, which involves subtraction of one blurred version of an image from a less blurred version of the image, was applied to separate close punctae. Thresholding parameters were applied manually for optimal punctae detection, and punctae were counted using the “Analyze Particles” function in *ImageJ*.

Corrected total cell fluorescence (CTCF) was calculated for both calretinin and tyrosine hydroxylase immunolabeling, which is defined as CTCF = Integrated fluorescence density per cell – (Area of selected cell x Mean fluorescence of background).

## Results

### Detection of *Tmprss3* mRNA by real-time PCR amplification

We first probed for the expression of *Tmprss3* mRNA by means of quantitative real-time PCR (qPCR). In the brainstem of mice, where several nuclei of the auditory pathway including the cochlear nucleus are located, no *Tmrpss3* mRNA transcripts were detected, both in young mice before the onset of hearing and in mature mice (Figure 1A). Next, we focused on the auditory periphery, where we sampled few cells of distinct types from P14-P19 mice to test for *Tmprss3* mRNA by Taqman-based qPCR. In the mouse organ of Corti, we found the strongest expression of *Tmprss3* mRNA in inner hair cells (IHCs) and outer hair cells (OHCs), and weaker expression in pillar cells (Figure 1B). In samples comprising of 3–12 neuronal cells of the Rosenthal’s canal each, we amplified a neuronal marker, *neurofilament heavy polypeptide* (*NF200*), and tested for a marker gene which is exclusively expressed in type II SGNs, *tyrosine hydroxylase* (*Th*) (Figure 1C). In all SGN samples, we found *NF200*, but not *Th* mRNA, indicating that these neurons were type I and not type II SGNs. In five independent neuronal samples, no *Tmprss3* mRNA was detected, in two other samples, we found very low levels of *Tmprss3* transcripts (Figures 1B, C).

### Expression of *Tmprss3* mRNA and protein in the mouse cochlea

By using RNAscope and combining it with immunohistochemistry we visualized the spatial expression of *Tmprss3* mRNA and protein in the mouse inner ear in a mature and an immature stage (Figure 2). In whole mounts of mature (P32) organs of Corti, we detected the highest levels of *Tmprss3* mRNA in IHCs and in cells of the spiral limbus, and lower levels in OHCs (Figure 2A, upper panel). On protein level, TMPRSS3 was most prominent in hair bundles of inner and outer hair cells and in the cuticular plate around IHCs (Figures 2A, B, upper panels).

In an earlier maturational stage at P10, we found the strongest *Tmprss3* mRNA signal in IHCs and in the spiral limbus surface (Figure 2A, lower panel, Figure 2B middle panel). Immunolabeling with the anti-TMPRSS3-antibody correlated well, indicating presence of TMPRSS3 protein at the cuticular plate and the hair bundles. The intensity of immunolabeling in stereocilia was less than at P32, indicating that TMPRSS3 protein levels rise during postnatal development, presumably around the onset of hearing. In the latter experiment we employed tissue of heterozygous littermates (*Tmprss3*^ + ⁣/−^) from *Tmprss3*-knock-out (*Tmprss3*^–/–^) animals, which we used to test for the specificity of TMPRSS3 immunolabeling (Figure 2B, lower panel). Importantly, we confirmed the absence of TMPRSS3-immunolabeling in hair cell stereocilia from *Tmprss3*^–/–^ animals at P10, which is shortly before the hair cells degenerate in the knock-out animals (Fasquelle et al., 2011; Figure 2B).

**FIGURE 3:**
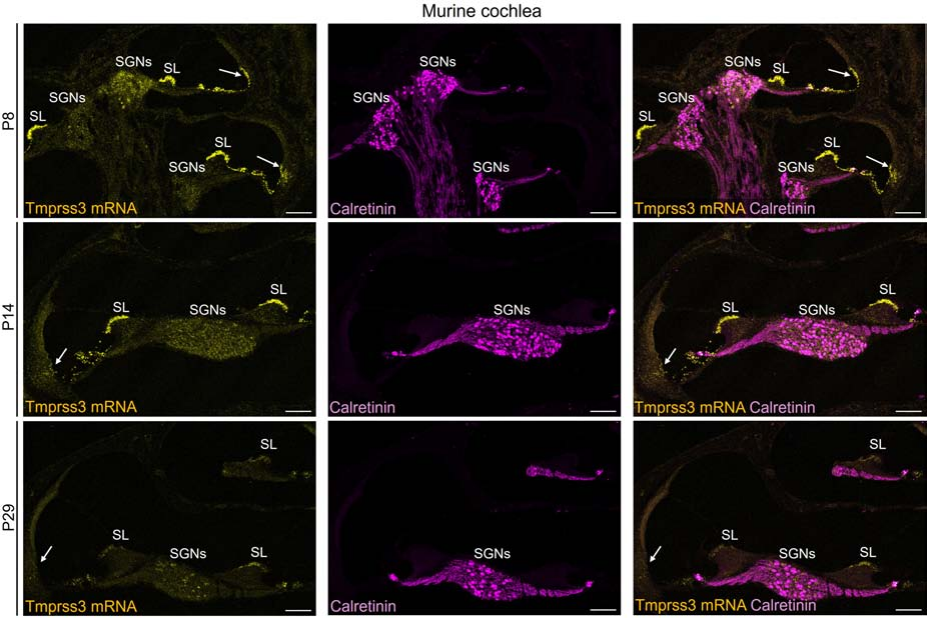
Representative images of *Tmprss3* mRNA via RNAscope detection (yellow) and calretinin immunostaining (magenta) in different developmental stages of the murine cochlea. Overview of a mid-modiolar cryosections of the cochlea from wildtype mice aged P8 **(upper)**, P14 **(middle)** and P29 **(lower panel)**. *Tmprss3* mRNA is expressed in both inner and outer hair cells, in supporting cells, in the spiral limbus (SL), cells of lateral wall, and in SGNs. Calretinin immunostaining labels hair cells and most of the type I SGNs. Scale bars: 100 μm.

**FIGURE 4:**
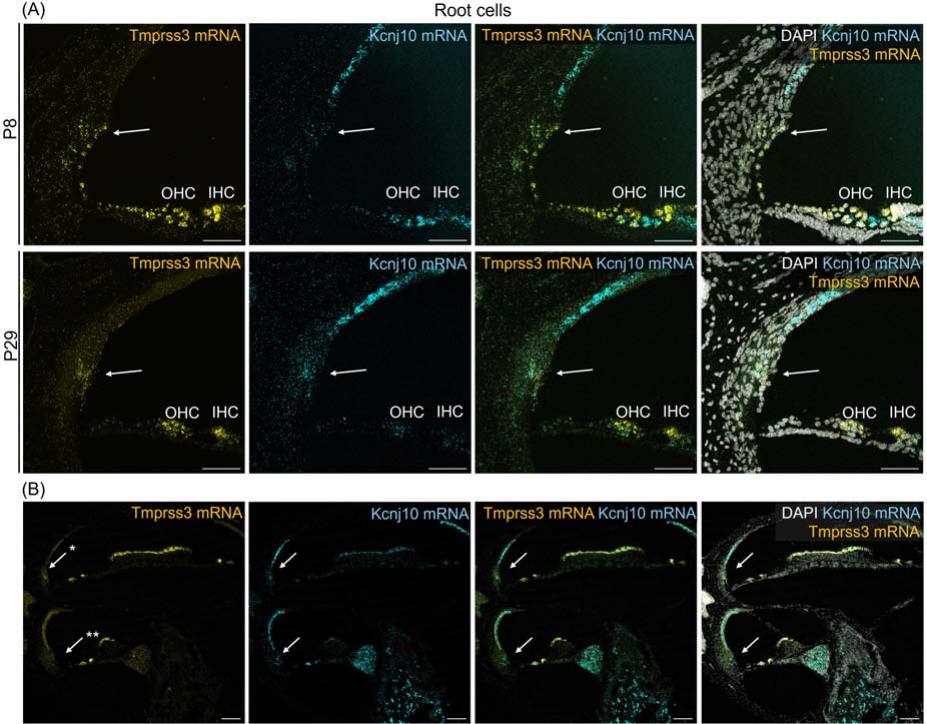
Representative images of an RNAscope experiment to visualize *Tmprss3* mRNA expression (yellow) in the lateral wall. To identify root cells, RNAscope was employed for detecting *Kcnj10* mRNA (blue). DAPI staining of cellular nuclei (white), was added to illustrate the tissue structure. **(A)** Root cells (arrows) of the lateral wall, positive for *Kcnj10* mRNA, from a mid-modiolar cochlear cryosection from a wildtype premature (P8) and mature (P29) mouse cochlea, also express *Tmprss3* mRNA. Scale bars: 50 μm. **(B)** *Tmprss3* mRNA expression in root cells is higher in apical turns (*) compared to midbasal turns (**). Scale bars: 100 μm. Arrows: root cells; IHC, inner hair cells; OHC, outer hair cells.

**FIGURE 5:**
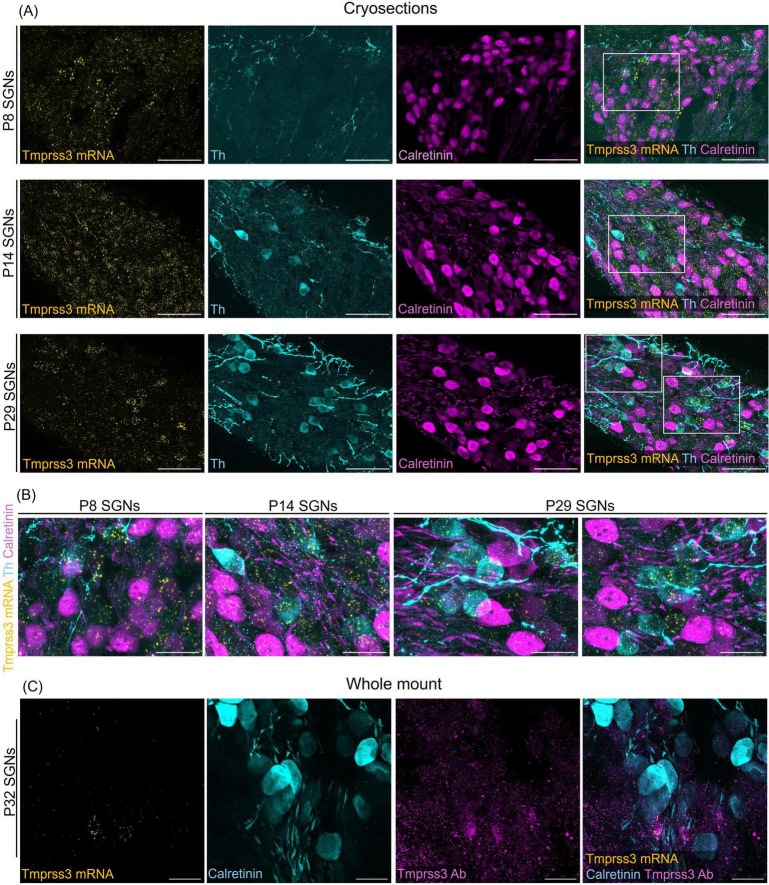
*Tmprss3* expression in type I and II SGNs at different developmental stages. **(A)** Representative image of modiolar cryosection displaying *Tmprss3* mRNA expression (yellow) in SGNs in type I (Calret +) and type II (Th +) SGNs at the age of P8 (upper), P14 (middle) and P29 (lower panel). While at P8 and P14, *Tmprss3* mRNA is found rather uniformly distributed across the Rosenthal’s canal, the mRNA of *Tmprss3* becomes restricted to few cells at P29, which were typically Th + cells. Scale bars: 50 μm. **(B)** Higher magnification images from insets in A, Scale bars: 20 μm. **(C)** TMPRSS3 immunostaining (magenta) and mRNA labeling with RNAscope (yellow) in SGNs at age P32 in a wholemount spiral ganglion, combined with calretinin immunostaining (blue) for type I SGNs. Scale bars: 20 μm.

We next assessed the cellular levels of *Tmprss3* mRNA in cochlear cryosections by means of RNAscope, which allowed us to analyze expression across different turns (Figure 3), in the stria vascularis (Figure 4), and to evaluate expression in different SGN types (Figure 5). We chose P8, P14, and P29 to examine *Tmprss3* expression before, around, and after the onset of hearing and SGN differentiation, respectively (Figure 3). *Tmprss3* mRNA was detected in certain cells of the organ of Corti at all developmental stages and in all cochlear turns. Specifically, we found *Tmprss3* mRNA in inner and outer hair cells, supporting cells, and in the spiral limbus at postnatal day 8 (Figure 3, upper panels). Around the onset of hearing at P14, the cellular expression pattern remained similar (Figure 3, middle panel). In sections from the mature cochleae (P29), the RNAscope signal for mRNA expression appeared generally weaker, but was still present in inner and outer hair cells (Figure 3, lower panel). Additionally, *Tmprss3* mRNA was found in the cells of spiral limbus, but also with less intensity than earlier in development (SL, Figure 3). Furthermore, *Tmprss3* mRNA was detected in the *Kcnj10* mRNA-positive root cells of the spiral ligament both at P8 and P29 (Figure 4, arrows), but not in any of the cell types of stria vascularis (visualized by DAPI staining of cell nuclei). Notably, *Tmprss3* mRNA levels appeared to be stronger in root cells of the apical turns compared to midbasal/basal turns (Figure 4B).

To distinguish SGN types, we immunolabeled type II SGNs for tyrosine hydroxylase (Th + cells) and type I SGNs for calretinin, also called calbindin2 (Calret + cells) in cochlear cryosections after visualizing *Tmprss3* mRNA with RNAscope (Figure 5). According to Shrestha et al. (2018), calretinin mRNA is expressed in all type Ia, all type Ib and in the majority of type Ic SGNs. At P8, the punctuate signal representing *Tmprss3* mRNA was localized throughout the cells in Rosenthal’s canal, including Calret + and Th + cells, even though the latter are very sparse and only weakly labeled for Th at this developmental stage. Similarly, around the onset of hearing (P14), *Tmprss3* mRNA expression was not restricted to any particular cell type, even though the SGNs were more differentiated at this stage and the Th levels and numbers of Th + cells had increased. In the mature inner ear, at P29, we observed an altogether lower signal of *Tmprss3* mRNA in most cells of the Rosenthal’s canal but a significantly higher *Tmprss3* expression in Th + cells (Figures 5A, B). Furthermore, we confirmed that mRNA expression of *Tmprss3* also leads to protein translation using an anti-TMPRSS3 immunostaining, which labeled *Tmprss3*-RNAscope positive cells (Figure 5C). The results indicate that TMPRSS3 is broadly expressed in SGNs in early maturational stages but is downregulated in type I SGNs, and that TMPRSS3 expression increases in type II SGNs in the mature cochlea.

### Quantitative analysis of *Tmprss3* mRNA expression in the Rosenthal’s canal

**FIGURE 6:**
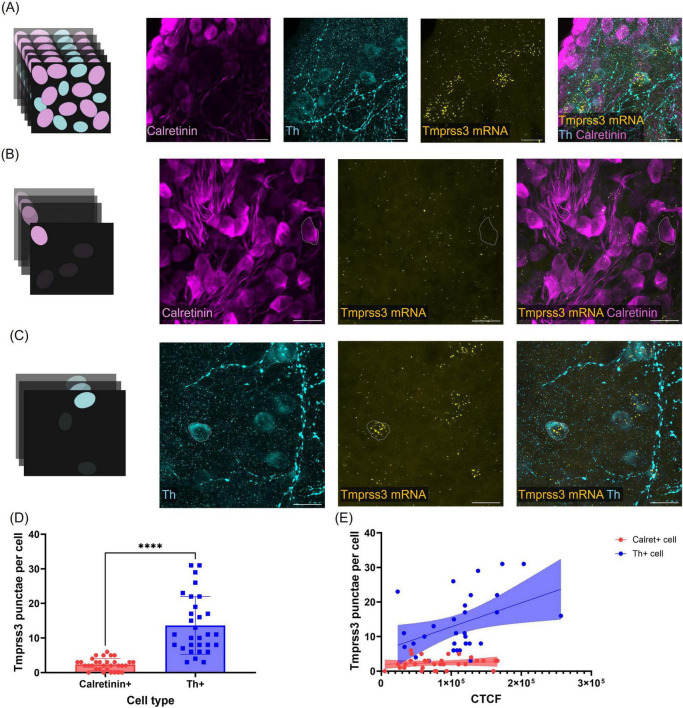
Quantification of *Tmrpss3* mRNA in type I and type II SGNs. **(A)** Maximum projection of a wholemount organ of Corti stained for *Tmprss3* mRNA (yellow), calretinin immunolabeling (magenta) to mark type I SGNs, and Th immunolabeling to indicate type II SGNs (blue), Scale bars: 20 μm. **(B)** Maximum projection of few optical sections to assess *Tmprss3* mRNA punctae in one outlined Calret + cell. **(C)** Maximum projection of few (other) optical cochlear sections to quantify *Tmprss3* mRNA punctae in one outlined Th + cell. **(D)** *Tmprss3* mRNA puncta per cell in Calret + and Th + SGNs. Unpaired *t*-test, ****p < 0.0001. **(E)** *Tmprss3* punctae counts were plotted against the fluorescence (corrected total cell fluorescence, CTCF) of calretinin (red) or Th (blue), respectively. *Tmprss3* mRNA punctae positively correlated with Th fluorescence, but not with calretinin fluorescence: Th + cells: *r* = 0.4261, *P* = 0.0189, Calret + cells: *r* = 0.1169, *P* = 0.5310.

We quantified the expression pattern of *Tmprss3* mRNA in the two distinct cell types in the Rosenthal’s canal, Calret + type I and Th + type II SGNs (Figure 6). *Tmprss3* mRNA punctae were counted in individual cells outlined according to the respective immunolabeling, in maximum projections comprising only the optical sections which covered the respective cells (Figures 6A–C). Again, we found an enrichment of *Tmprss3* mRNA in type II SGNs (Figure 6D), which make up roughly 5% of the total SGN population (Spoendlin, 1969) and are presumed to be involved in auditory nociception, but play no major role for encoding auditory information (Flores et al., 2015). Furthermore, the *Tmprss3* mRNA puncta count positively correlated with the tyrosine hydroxylase expression (Figure 6E, *r* = 0.4261, *P* = 0.0189). In contrast, RNAscope punctae were significantly fewer in cell bodies positive for calretinin and did not correlate with the expression levels of calretinin (Figure 6E, *r* = 0.1169, *P* = 0.5310).

## Discussion

In this study, we characterize the expression pattern of *Tmprss3* in the mouse cochlea using the RNAscope technique for mRNA *in situ* hybridization, a TaqMan-probe based quantitative real-time PCR, and fluorescence immunohistochemistry with an antibody validated on knock-out tissue. Our data confirm strong *Tmprss3* expression in the organ of Corti, particularly in hair cells, where the protein localized specifically to the hair bundles. In Rosenthal’s canal, *Tmprss3* mRNA expression was limited to very low levels in mature type I SGNs. Notably, we found substantial *Tmprss3* mRNA in type II SGNs, which correlated with TMPRSS3 immunolabeling, indicating that not only mRNA but also TMPRSS3 protein is expressed in type II SGNs.

Our findings here corroborate and provide visual evidence to recent data from RNAseq studies that also reported *Tmprss3* expression to be enriched in type II SGNs of mouse or human cochlea (Chen et al., 2022; Shrestha et al., 2018; Sun et al., 2018).

Previous studies described *Tmprss3* expression in both the organ of Corti and in SGNs in rodents (Guipponi et al., 2002, 2008). In agreement with our findings, Guipponi et al. (2002) found *Tmprss3* mRNA in cell bodies of Rosenthal’s canal in P5 mice, but no later developmental stages were analyzed at that time. In a follow-up study, PCR confirmed *Tmprss3* expression in SGNs at P5, and immunohistochemistry seemed to display a widespread TMPRSS3 expression in all SGNs of a mature cochlea (Guipponi et al., 2008). Fasquelle et al. (2011) reported a faint hybridization signal for *Tmprss3* mRNA in Rosenthal’s canal of a P7 mouse which became constrained to few cells at P11. Although these cells were not further characterized at the time, the sparse cellular labeling might be consistent with *Tmprss3* mRNA expression in type II SGNs. Consistently, our qPCR hardly captured *Tmprss3* cDNA in several samples of 3–12 randomly selected type I SGNs at P14-19. In addition, our quantitative RNAscope analysis argues against a substantial expression of *Tmprss3* in mature type I SGNs. In early postnatal maturational stages, when SGNs just begin to differentiate into type I and II, we detected *Tmprss3* mRNA in most cell bodies in the Rosenthal’s canal. Whether this expression is of functional relevance for SGN development and health still needs to be determined. Since the levels of *Tmprss3* mRNA declines in type I SGNs during maturation, we hypothesize that the weak RNAscope signal in more mature type I SGNs reflects residual or declining expression. In contrast, the level of *Tmprss3* mRNA scaled with Th expression levels marking type II SGNs, and immunolabeling detected TMPRSS3 protein in cells displaying *Tmprss3*-RNAscope signals. A more elaborate analysis of TMPRSS3 immunolabeling in Rosenthal’s canal was challenged by (i) the weakness of the antibody, which bound its target only when applying the immunostaining protocol after the RNAscope protocol (as in all our experiments), and (ii) because the anti-Th antibody used to mark type II SGNs was raised in the same host animal as the anti-TMPRSS3 antibody, such that both antibodies could not be applied in parallel.

Considering the high stringency and specificity of the methods applied in our study, the previously described TMPRSS3 expression in mature type I SGNs might be doubted, as the antibody used in Guipponi et al. (2008) was not validated in *Tmprss3*-knock-out tissue and no analysis of mRNA expression in mature tissue has been performed. Congruent with our findings, Liu et al. (2018) detected TMPRSS3 protein in the mature human organ of Corti but reported only unspecific staining in cell bodies of spiral ganglia and no immunolabeling in nerve fibers. Moreover, similar to our results, they described TMPRSS3 to localize to stereocilia of human auditory hair cells, suggesting a conservation across species and pointing to a potential functional role for TMPRSS3 at this structure.

We also identified *Tmprss3* expression in *Kcnj10* mRNA-positive root cells of the spiral ligament at P8 and P29, in agreement with RNAseq data reporting the presence of *Tmprss3* mRNA in root and spindle cells (Koh et al., 2023; Gu et al., 2020). Interestingly, the *Tmprss3*-RNAscope signal was highest in the apical turn of cochlea (Figure 4). Contrary to previous studies reporting *Tmprss3* expression in stria vascularis in P5 rodents (Guipponi et al., 2002), we found no signal in marginal, intermediate or basal cells of stria vascularis in either the immature (P8) or mature cochlea (P29). The expression of *Tmprss3* in the spiral ligament might relate to its role in regulating the timing of the increase in endocochlear potential, which was found to occur earlier in TMPRSS3 deficient cochleae (Shearer et al., 2025).

In light of first attempts trying to preserve cochlear function in *Tmprss3*-deficient or mutant animal models by gene therapy, our data indicate that cellular targeting of the vectors transducing the coding sequence likely needs to be refined toward hair cells, pillar cells, root cells and type II SGNs. The previous strategies forced to express the *Tmprss3* coding sequence in mature type I SGNs, which might have contributed to adverse effects like the observed toxicity of murine *Tmprss3* cDNA (Du et al., 2023), or deleterious effects of overexpression in inner ear tissue (Aaron et al., 2023). However, if the transient expression of *Tmprss3* during early development in SGNs would be crucial for long-term SGN function and health, any postnatal gene therapy in humans, targeting a mature cochlea, would be unlikely to fully restore SGN function.

Considered to be one of the most advanced neural prosthetics, cochlear implants have unquestionably helped improve the life quality of individuals with severe-to-profound forms of hearing loss. Several factors contribute to the postoperative success of CIs including the age of implantation, genotype, and underlying pathophysiology (Shearer et al., 2017). In DFNB8/10 patients, CI outcomes were reported to be poor (Shearer et al., 2017; Tropitzsch et al., 2023), mixed (Lee et al., 2020, 2023; Cottrell et al., 2023; Colbert et al., 2024) or favorable (Weegerink et al., 2011; Miyagawa et al., 2015; Holder et al., 2021; Carlson et al., 2023). A meta-analysis by Chen et al. (2022) concluded that the CI outcomes in individuals with TMPRSS3 mutations are comparable to individuals from other hearing loss cohorts. Additionally, Colbert et al. (2024) reported in their extensive cohort study that poor performance correlates with higher age at implantation.

According to the “spiral ganglion hypothesis,” pathogenic mutations affecting the health and function of the spiral ganglia lead to poorer CI outcomes than those impairing sensory epithelia (Shearer et al., 2017). The spiral ganglion hypothesis is often simplified toward the idea that genes that are expressed in the SGNs lead to, when mutated, poor CI performance. Our data demonstrate minimal or no expression of *Tmprss3* in mature type I SGNs, which are critical for auditory stimulus transmission and the proper functioning of CIs. We thus hypothesize that TMPRSS3 expression in proximity to type I SGNs is critical for function and maintenance of type I SGNs. Alternatively, the notable expression of *Tmprss3* in immature neuronal cells of the Rosenthal’s canal could potentially affect the differentiation and health of the developing type I SGNs, impacting their function and long-term survival in adulthood. Notably, in otic organoids deficient for TMPRSS3 a severe decay in electrical excitability was unraveled in SGN-like cells, although also in this model *Tmprss3* expression was again restricted to type II SGN-like cells (unpublished results). Furthermore, we did not detect any *Tmprss3* expression in the brainstem, such that an expression in cochlear brainstem nuclei can be ruled out as a reason for low CI performance.

The *transmembrane serine protease 3* gene exhibits a serine protease domain on the extracellular (or intra-luminal) partition of the protein, but the targets of the protease have not been identified to date. Notably, brain-derived neurotrophic factor (BDNF) and neurotrophin 3 (NT-3), which play a key role for the developing innervation of the inner ear (Fritzsch et al., 1999; Singer et al., 2014), are activated by the proteolytic cleavage of a pro-form of the molecules. Pro-BDNF plays distinct roles from those of BDNF, and its high presence in the early postnatal brain suggests that it acts in axonal growth, synaptic maturation and pruning. Processing by serine proteases, however, leads to the predominant release of BDNF which has well-known neurotrophic and neuroprotective roles (Yang et al., 2009). A different study found pro-BDNF to induce synaptic pruning at neuromuscular junctions in early postnatal rodents (Je et al., 2013). Notably, (pro-)BDNF is highly expressed in the hair cells of the organ of Corti (gEAR database, Kolla et al., 2020). Combined, these studies suggest that TMPRSS3 may process the pro-neurotrophins, thereby influencing the development and health of the inner ear. While this needs to be experimentally tested, a systematic screen to detect targets of the TMPRSS protein family might point to roles in different functional processes and will help to understand the pathophysiological mechanisms in TMPRSS deficiency. Interestingly, TMPRSS2 was recently reported to cleave α-tectorin, which is a major component of the tectorial membrane (Niazi et al., 2024). In our study, we also report the expression of *Tmprss3* in the region of interdental cells of the spiral limbus, which is the location where TMPRSS2 cleavage of α-tectorin was observed. Moreover, *Tmprss1* deficiency was found to cause profound hearing loss, with an abnormally developed tectorial membrane and morphological defects in Rosenthal’s canal (Guipponi et al., 2007).

Our data, which is in agreement with other work studying mRNA expression in adult SGNs, indicate that type I SGNs do not substantially express TMPRSS3. Since in absence of TMPRSS3, the electrical excitability of human SGNs is reduced, rodent SGNs degenerate, and SGN-like cells from otic organoids display reduced ionic currents, TMPRSS3 does play a role for the health of adult SGNs despite not being expressed there. We hypothesize the effect on SGN health to originate either from an impact of its early developmental expression in SGNs, or an indirect downstream or cascade effect of TMPRSS3 action, being expressed from other cochlear cells. Further studies investigating the function of TMPRSS3 in the cochlea, particularly its targets, are needed to understand its associated pathophysiology and revisit treatment directions for DFNB8/10 individuals.

## Data Availability

The original contributions presented in this study are included in this article/supplementary material, further inquiries can be directed to the corresponding author.

## Footnotes

1
https://hereditaryhearingloss.org
